# The Wilbrand’s knee does not exist in the optic chiasm

**DOI:** 10.1007/s00381-018-3969-5

**Published:** 2018-09-08

**Authors:** Andrzej Grzybowski, Piotr Kanclerz

**Affiliations:** 1 Department of Ophthalmology, University of Warmia and Mazury, Olsztyn, Poland; 2 Foundation for Ophthalmology Development, Institute for Research in Ophthalmology, Gorczyczewskiego 2/3, 60-554 Poznan, Poland; 3 Private Practice, Gdańsk, Poland

Dear Editor:

We have read the article by Costea and associates; however, we believe that some discussion is required [1]. The authors review the history of the optic chiasm from antiquity to the twentieth century. It is presented that within the optic chiasm, the nervous fibers from the lower retinal quadrants loop forward into the termination of the opposite optic nerve before passing back into the optic tract (named anatomically the *Wilbrand’s knee*).`

Indeed, the *Wilbrand’s knee* appears in the majority of current textbooks of ophthalmology and neuro-ophthalmology, and damage to it was previously assumed to be responsible for junctional scotoma. However, *Wilbrand’s Knee* is an artifact, and does not exist in the normal primate optic chiasm [2]. Horton demonstrated that optic nerve fibers cross the optic chiasm without entering the contralateral optic nerve [3]. The *Wilbrand’s knee* forms in long-term after monocular enucleation, presumably from shrinkage of the optic chiasm caused by atrophy of fibers from the enucleated eye. Therefore, the anterior chiasmal syndrome occurs from combined compression of the optic chiasm and one or both optic nerves, and has a limited localizing value.
